# Dexmedetomidine Pharmacokinetics in Neonates with Hypoxic-Ischemic Encephalopathy Receiving Hypothermia

**DOI:** 10.1155/2020/2582965

**Published:** 2020-02-25

**Authors:** Ryan M. McAdams, Daniel Pak, Bojan Lalovic, Brian Phillips, Danny D. Shen

**Affiliations:** ^1^Department of Pediatrics, University of Wisconsin School of Medicine and Public Health, Madison, WI, USA; ^2^Department of Pharmacy, Seattle Children's Hospital, Seattle, WA, USA; ^3^Clinical Pharmacology Sciences, Eisai Inc., Woodcliff Lake, NJ, USA; ^4^Pharmacokinetics Laboratory, Department of Pharmaceutics, School of Pharmacy, University of Washington, Seattle, WA, USA

## Abstract

Dexmedetomidine is a promising sedative and analgesic for newborns with hypoxic-ischemic encephalopathy (HIE) undergoing therapeutic hypothermia (TH). Pharmacokinetics and safety of dexmedetomidine were evaluated in a phase I, single-center, open-label study to inform future trial strategies. We recruited 7 neonates ≥36 weeks' gestational age diagnosed with moderate-to-severe HIE, who received a continuous dexmedetomidine infusion during TH and the 6 h rewarming period. Time course of plasma dexmedetomidine concentration was characterized by serial blood sampling during and after the 64.8 ± 6.9 hours of infusion. Noncompartmental analysis yielded descriptive pharmacokinetic estimates: plasma clearance of 0.760 ± 0.155 L/h/kg, steady-state distribution volume of 5.22 ± 2.62 L/kg, and mean residence time of 6.84 ± 3.20 h. Naive pooled and population analyses according to a one-compartment model provided similar estimates of clearance and distribution volume. Overall, clearance was either comparable or lower, distribution volume was larger, and mean residence time or elimination half-life was longer in cooled newborns with HIE compared to corresponding estimates previously reported for uncooled (normothermic) newborns without HIE at comparable gestational and postmenstrual ages. As a result, plasma concentrations in cooled newborns with HIE rose more slowly in the initial hours of infusion compared to predicted concentration-time profiles based on reported pharmacokinetic parameters in normothermic newborns without HIE, while similar steady-state levels were achieved. No acute adverse events were associated with dexmedetomidine treatment. While dexmedetomidine appeared safe for neonates with HIE during TH at infusion doses up to 0.4 *μ*g/kg/h, a loading dose strategy may be needed to overcome the initial lag in rise of plasma dexmedetomidine concentration.

## 1. Introduction

Worldwide, hypoxic-ischemic encephalopathy (HIE) is a leading cause of neonatal brain injury and neonatal mortality [1]. In high-income countries, therapeutic hypothermia (TH) is the standard therapy to mitigate brain damage in newborns with HIE. While TH appears to increase survival without increasing major disability in survivors, 1 in 4 neonates with moderate-to-severe HIE die despite receiving TH [2]. Newborns with moderate-to-severe HIE often have multiorgan failure and may demonstrate seizures, respiratory failure, and cardiovascular instability. They commonly receive morphine for sedation and to prevent shivering [3]. However, efficacy of morphine as an adjunctive therapy during TH has not been evaluated in clinical trials. Morphine also requires dosing adjustments to avoid toxicity due to altered pharmacokinetics during TH [4]. Safety concerns with morphine include short-term side effects such as depressed ventilation and questionable effects on long-term neurodevelopmental outcomes [5–7].

Dexmedetomidine, a highly lipophilic *α*_2_-adrenergic receptor agonist metabolized in the liver and primarily excreted by the kidney, may be a promising alternative to morphine for newborns with HIE treated with TH. Dexmedetomidine provides sedation and prevents shivering but does not suppress ventilation [8–11]. Information on the safety and efficacy of dexmedetomidine in infants is limited, and the drug is not approved for this age group by the Food and Drug Administration. However, dexmedetomidine is increasingly being used off-label for sedation in infants [11–14]. Considerable variation in dexmedetomidine dosing has been reported in studies involving infants, ranging from 0.05 to 1 *μ*g/kg for loading dose and from 0.1 to 2.5 *μ*g/kg/h for continuous infusion [12, 13, 15–17]. Although pharmacokinetic-pharmacodynamic data to inform dexmedetomidine dosing in neonates are limited, available studies have demonstrated decreased dexmedetomidine clearance in infants compared to adults [13, 15, 17].

Currently, dexmedetomidine pharmacokinetic data in neonates with HIE receiving hypothermia are lacking and pharmacokinetic data from cooled neonatal animals [14, 18] are insufficient to guide clinical practice. Neonates with HIE receiving TH often have altered pharmacokinetics and dosing needs [4, 19, 20]. In the setting of neonatal HIE, whether hypoxia-induced liver injury and hypothermia alter dexmedetomidine pharmacokinetics remains unknown. Therefore, the overall objective of the current prospective clinical study is to evaluate the pharmacokinetics and safety of dexmedetomidine in neonates with HIE receiving TH, which will inform the design of future clinical efficacy trials in this population. The pharmacokinetic portion of the trial is aimed primarily on the question of whether dexmedetomidine pharmacokinetics in HIE neonates undergoing TH differ from those reported for normothermic, non-HIE neonates and secondarily to provide initial pharmacokinetic data in designing a comprehensive population PK-PD study later in a larger patient population.

## 2. Methods

### 2.1. General Study Design

This study received Seattle Children's Hospital Institutional Review Board approval and was registered with the US Food and Drug Administration (Investigational New Drug 127874) and ClinicalTrials.gov (NCT02529202). Informed parental consent was obtained for each neonate enrolled. This is a phase I, single-center, prospective, open-label clinical study of dexmedetomidine pharmacokinetics and safety in neonates with HIE receiving TH. This study was conducted in Seattle Children's Hospital level IV neonatal intensive care unit (NICU), a center with expertise in caring for outborn neonates with HIE and using TH.

### 2.2. Patients

The study population consisted of intubated neonates who were ≥36 weeks' gestational age, diagnosed with moderate-to-severe perinatal HIE, and treated with TH (target temperature 33.5°C) for a planned duration of 72 h. Neonates meeting inclusion and exclusion criteria were enrolled consecutively. Criteria for TH were similar to those in the CoolCap [21] or National Institute of Child Health and Human Development studies [22]. Intubated neonates could be extubated any time during the study period at the discretion of the neonatology team. Exclusion criteria included known chromosomal anomalies, cyanotic congenital heart defects, lack of an indwelling central intravenous line, neonates participating in another clinical trial, neonates who received dexmedetomidine prior to study enrollment, withdrawal of care being considered because of moribund condition, or a decision made to withhold full support.

### 2.3. Dexmedetomidine Administration

The study drug, dexmedetomidine hydrochloride injection (100 mg/mL base), was administered in a primed intravenous line via a computer-controlled infusion device programmed by trained NICU nurses. At the time of study, the Seattle Children's Hospital Pharmacy and Therapeutics Committee had established a dexmedetomidine dosing range of 0.2–1.2 *μ*g/kg/h in critical care areas. For the current study, a maximum dexmedetomidine dose of 0.4 *μ*g/kg/h was used, a dose that was well tolerated without significant adverse side effects based on our clinical experience with normothermic neonates. Given that all potential study candidates were outborn neonates, the window for enrollment was within the first 24 h after TH was started. After study enrollment, dexmedetomidine infusion was started at 0.2 *μ*g/kg/h, increased to 0.3 *μ*g/kg/h after one hour, then increased to 0.4 *μ*g/kg/h after 2.5 h, and maintained at that dose until the neonate had completed the 6 h rewarming period following 72 h of TH. After rewarming and once normothermia was reestablished, dexmedetomidine was discontinued without weaning the dose. Adjustment of the dexmedetomidine dose (i.e., decreasing or holding the dose) during TH was based on the discretion of the treating clinical team. Open-label morphine was available for rescue analgesia or to prevent shivering as per the discretion of the medical team. The incidence, dose, and duration of treatment with adjunctive sedation medications were recorded.

### 2.4. Pharmacokinetic Sampling

The blood-sampling schedule was designed to capture the rise in blood dexmedetomidine concentration during the titration phase, the maximum or plateau level reached during maintenance infusion, and the washout kinetics after discontinuation. A total of 17 blood samples (0.3 mL whole blood each) were collected from each neonate's central line, which was in three periods: a single baseline sample (1) prior to dexmedetomidine initiation, (2) during dexmedetomidine infusion at 15, 30, 60, 120, 180, and 270 min and 6, 10, 24, and 48 h after start of infusion, and (3) at 0, 1, 2, 4, 18, and 42 h after dexmedetomidine discontinuation. The dexmedetomidine infusion occurred in a separate intravenous line from the neonate's central line, where blood samples were drawn. Over the 5-day (120 h) study period, a total blood volume of up to 5.1 mL (17 × 0.3 mL) was withdrawn for research purposes. Blood samples were collected in 1 mL sterile cryogenic vials (Globe Scientific CryoClear™ vials, Paramus, New Jersey); plasma was separated by centrifuging sample vials at 1,200 rpm for 15 min, then frozen, and stored at −80°C in 1 mL sterile cryogenic vials for subsequent analysis.

### 2.5. Analytical Quantification

A sensitive liquid chromatography-tandem mass spectrometry method was developed for analyzing dexmedetomidine concentration in 100 *μ*L volume of plasma. Dexmedetomidine and the internal standard medetomidine-d_3_ (Toronto Research Chemicals, Toronto, ON) were extracted from plasma samples using Bond Elut Certify cartridges (Agilent Technologies, Santa Clara, CA). For chromatographic separation, extracts were injected onto a Restek Ultra Aqueous C18 column, 2.1 mm × 200 mm × 5 *μ* (Bellefonte, PA), operated with an isocratic mobile phase consisting of a 45 : 55 mix of 0.1% formic acid in water (mobile phase A) and methanol (mobile phase B) at a flow rate of 0.25 mL/min and temperature of 35°C. For quantification, the eluent was directed to an AB Sciex 6500 Q-Trap triple-quadrupole mass spectrometer using an electrospray ion source operating in the positive ion mode. Transitions for single reaction monitoring of dexmedetomidine and internal standard are detailed in Supplementary [Supplementary-material supplementary-material-1]. Quality control samples were run with each batch of plasma samples. The lower limit of quantitation was 1 ± 0.6 pg/mL. The interday coefficient of variation was <7% for both the low and high QCs.

### 2.6. Pharmacokinetic Analysis

Two types of pharmacokinetic analyses were performed, i.e., an initial noncompartmental (descriptive) analysis followed by naïve pooled and initial population-based compartmental modeling.

Plasma concentration data collected during and after discontinuing dexmedetomidine infusion in each of the 7 HIE newborns were subject to noncompartmental analysis. The highest observed plasma dexmedetomidine concentration (*C*_max_ (pg/mL)) and time of its occurrence (*T*_max_ (h)) during dexmedetomidine infusion were noted. The decline in plasma concentration with time after discontinuing the dexmedetomidine infusion was fitted to either a monoexponential or biexponential function to yield an estimate of terminal exponential rate constant (*λ*_z_ (h^−1^)) using the numerical module of SAAM II (v2.0; University of Washington, Seattle, WA). Area under the plasma concentration-time curve from time zero (i.e., infusion start time) to time infinity (AUC_0–∞_ (pg/mL·h)) was calculated by a linear trapezoidal rule up to the last sampling time, and area beyond the last observed concentration (*C*_last_) was extrapolated by *C*_last_/*λ*_z_. Plasma clearance (CL_cor_ (L/h/kg)) was calculated by the total dose infused/AUC_0–∞_; the infused dose was corrected for sorptive loss of dexmedetomidine to microbore tubing (see below). Area under the first moment curve (AUMC_0–∞_ (pg/mL·h [2])) was also calculated by a linear trapezoidal rule up to the last sampling time, and area beyond was extrapolated as the sum of *C*_last_·*t*_last_/*λ*_z_ and *C*_last_/*λ*_*z*_^2^. The mean residence time (MRT (h)) was calculated from the ratio of AUMC_0–∞_ and AUC_0-∞_ corrected for infusion time (i.e., *T*_inf_/2). Steady-state distribution volume (*V*_ss_) was further calculated from the product of MRT and CL_cor_.

The available set of dexmedetomidine pharmacokinetic data for the 7 HIE newborns was then analyzed by nonlinear mixed-effects (population) modeling. The analysis was performed using NONMEM software (version 7.4.1; ICON Development Solutions, Ellicott City, MD) using the first-order conditional estimation method with residual variability (*η*–*ε*) interaction (FOCEI). Given the small dataset, a naïve pooled analysis was initially performed and subsequently expanded to include a random-effect parameter. The objective function value (OFV, the −2 log likelihood) was used to judge goodness of fit when comparing nested models. While the difference in model OFVs (ΔOFV) is often assumed to approximate a chi-squared distribution, the assumption may not apply to the current small dataset; hence, no critical ΔOFV for a given level of probability significance was assumed. In addition to consideration of standard nonlinear mixed-effects diagnostics, the model condition number (ratio of the largest to the smallest eigenvalue of the variance-covariance matrix) served as a guide against overparametrization (i.e., models with a high degree of collinearity amongst model parameters); only models with condition number below 1000 were considered acceptable [23].

Two additional adjustments to the population model were implemented. First, as dictated by the clinical circumstance and as allowed under the study protocol, dexmedetomidine infusion deviated from protocol design in three of the 7 subjects; hence, dosing input consisting of escalating, body weight-normalized constant-rate (0-order) infusion was individually specified according to the record of dexmedetomidine infusion. Furthermore, the input rate of dexmedetomidine was corrected for loss due to sorption to microbore tubing as described in a later section. Additionally, dexmedetomidine concentrations in 14.7% of samples, those primarily collected early on during dexmedetomidine infusion, fell below the lower limit of quantification likely due to sorptive loss and slow accumulation. Therefore, we examined the impact of censoring of dexmedetomidine pharmacokinetic data based on the “NONMEM M3 method” [24, 25].

Both one-compartment and two-compartment models were initially evaluated; the reasonableness and precision of the parameter estimates, as well as several graphical goodness-of-fit diagnostic plots, were considered. All covariates were analyzed as univariate predictors of dexmedetomidine clearance or distribution volume in a stepwise process; they included body weight, gestational and postmenstrual ages, serum alanine transferase (ALT), and maximum creatinine concentration (CrMax). Two physiologically relevant covariates on either clearance or distribution volume were also evaluated: duration of cooling prior to initiation of dexmedetomidine infusion and variation of body temperature over the course of study. It should be noted that addition of either of these covariates would result in changes in clearance or distribution volume over time; hence, this was an attempt to discern the presence of time-varying or nonstationary dexmedetomidine pharmacokinetics as a result of hypothermia and rewarming. A more empirical approach to modeling such time-varying pharmacokinetic processes was also attempted by allowing either exponential or linear change in clearance or distribution volume over time.

Pharmacokinetic data and the NONMEM code for the modeling analyses are available to other investigators upon request.

### 2.7. Infusate Sampling to Assess Microbore Tubing Dexmedetomidine Sorption

In 4 of the 7 cooled neonates with HIE, plasma dexmedetomidine concentration remained low or undetectable during the initial 1 to 3 hours of infusion, which led to the question of whether some portion of the dexmedetomidine dose may be lost through sorption to intravenous tubing during dexmedetomidine infusion. To test this possibility, infusates were serially collected in three mock infusion experiments performed with an intravenous pump and tubing setup identical to the setup used with all 7 cooled neonates with HIE in the study. An Alaris model 8100 infusion pump (CareFusion, San Diego, CA) driven by an Alaris model 8015 PC unit was coupled to a microbore tubing (213 cm in length) extension set (CareFusion) using MaxZero minibore extension set intravenous connectors (CareFusion).

For experiment 1, a time zero sample was taken from the dexmedetomidine syringe and 8 additional infusate samples were collected from the outflow of the microbore tubing at times corresponding to the clinical study time points through the first 6 h of dexmedetomidine infusion. For experiments 2 and 3, preinfusion samples were drawn from the dexmedetomidine syringe and immediately from the outflow after priming the microbore tubing. Serial collection of the outflow infusate was extended to 18 h and included 12 total time points to capture the sorptive loss beyond the titration phase and into the plateau phase during maintenance infusion. At the end of all three experiments, a sample of the infusate left in the syringe was collected immediately upon stopping the infusion pump. Any loss in dexmedetomidine while the infusate was held in the syringe or flowing through the microbore tubing was expressed as fraction of nominal concentration (FNC), which is the ratio of assayed concentration in the sampled infusate to the originally prepared concentration of 4 *μ*g/mL. FNC in the serially sampled outflow from the microbore tubing was plotted against time.

**Figure 1:**
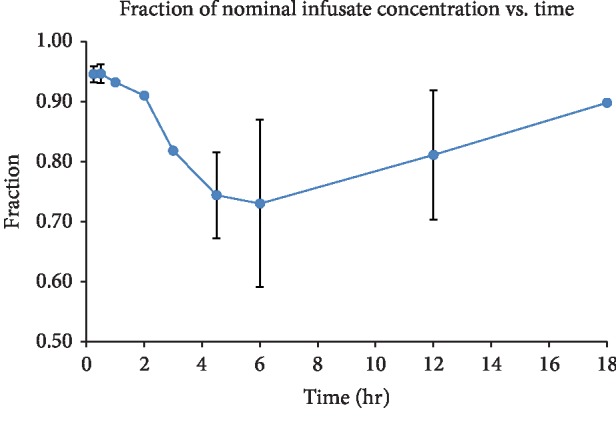
Sorptive loss of dexmedetomidine to IV microbore tubing assessed over an 18-h mock infusion experiment. The mean ± standard deviation of the fraction of nominal concentration (FNC) in the outflow infusate from 3 separate experiments is plotted against time.

In the noncompartment analysis, for each neonate, the actual dexmedetomidine dose delivered during successive intervals between blood sampling or between change in infusion rate and the next blood sampling was calculated by multiplying the intended infused dose (i.e., nominal infusion rate × interval duration) by the average FNC at the mid-time point as estimated by linear interpolation of the FNC vs. time plot up to the last time point and extrapolation to the time when sorptive loss stopped based on projection of the last two data points (Figure 1). The total delivered dexmedetomidine dose over the duration of TH and rewarming (i.e., *D*_cor_ or total dose corrected for sorptive loss to microbore tubing) was calculated by summing the corrected doses from all the successive sampling intervals. Clearance corrected for sorptive loss of infused dose to microbore tubing (CL, L/h/kg) was estimated by *D*_cor_/AUC_0–∞_.

In the naïve pooled and population compartmental modeling, dexmedetomidine infusion rates during successive intervals between blood samplings or between the time of a change in infusion rate and the next blood sampling were adjusted for sorptive loss using the F1 parameter in NONMEM, which was assumed to be the same for every individual. Values of F1 were derived from linear interpolation of the plot of the observed FNC over time (Figure 1) to yield FNC estimates at the mid-time points of successive sampling intervals. Also, the time of return to 100% FNC (i.e., ending of sorptive loss) was estimated to be 25.1 h by extrapolation of the last two data points at 12 and 18 h.

### 2.8. Clinical Assessments

Shivering was assessed and recorded using a modified version of the Bedside Shivering Assessment Scale described by Badjatia et al. that has been validated in brain-injured adults, but not neonates [26]. A 4-point scale was used, which rated shivering as *none*: no shivering noted (0), *mild*: shivering localized to the neck and/or chest only (1), *moderate*: shivering involves upper extremities, plus neck and chest (3), or *severe*: shivering involves upper and lower extremities, plus neck and chest (4). For any shivering episodes noted during TH, newborn shivering assessment scores were recorded along with the date, time, and duration of the episode and whether morphine was given.

For each neonate in the study, Neonatal Pain, Agitation, and Sedation Scale (N-PASS) scores were evaluated prior to and during dexmedetomidine exposure to determine sedation and analgesia effectiveness. The N-PASS tool, which has been validated in neonates [27], uses 5 assessment criteria (crying/irritability, behavior/state, facial expression, extremities/tone, and vital signs); each criterion is graded 0, –1, or –2 for sedation and 0, 1, or 2 for pain/agitation. An N-PASS total score >3 was thought to reflect significant pain or agitation, at which point supplemental sedation or analgesia therapy could be administered at the discretion of the medical team.

### 2.9. Safety Evaluations

Safety evaluations included continuous heart rate and blood pressure monitoring, laboratory measurements including complete blood count, basic metabolic panel, and liver function tests, and monitoring for any adverse events or serious adverse events. Adverse events included bradycardia (<70 beats per min), hypotension (<32 mm Hg mean arterial pressure), atrial fibrillation and renal failure (creatinine > 1.4 mg/dL), acute respiratory failure (requiring mechanical ventilation), or a central line-associated blood stream infection during dexmedetomidine infusion. Serious adverse events included severe cardiorespiratory decompensation (e.g., sinus arrest) or death related to dexmedetomidine infusion. An Internal Safety Monitoring Committee reviewed the NICU clinical course of all enrolled neonates; their review started after enrollment of the first 4 neonates.

### 2.10. Statistical Analyses

Group data are presented as means ± standard deviations. Mean estimates of descriptive, noncompartmental pharmacokinetic parameters for our cohort of cooled newborns with HIE were compared to a similar set of parameters for normothermic, full-term neonates without HIE reported by Chrysostomou et al. [15] using unpaired, 2-tailed *t*-tests for samples with unequal variance. Additional comparisons were also conducted with two other parallel sets of individual-level (adjusted for covariates of postmenstrual age and body weight), descriptive pharmacokinetic parameters that were generated from population compartmental models reported by Greenberg et al. [13] and Potts et al. [28] based upon dexmedetomidine pharmacokinetic data collected from mixed populations of neonates, infants, and young children. All calculations were performed using the statistical programming language R [29].

## 3. Results

In total, 7 HIE neonates treated with TH were enrolled in the study. Five other neonates were identified but were not included in the study, three whose parents declined enrollment and two whom the neonatology team deemed likely to have early withdrawal of care due to severe HIE. All infants were cooled to the target temperature of 72 h using whole body cooling; target cooling temperatures were achieved at 5.2 ± 1.6 h after delivery (range: 3.7 to 8.5 h). TH had already begun in all 7 neonates by the time parental consent was obtained; dexmedetomidine infusions were initiated at an average of 14 ± 6.5 h after TH was started (i.e., pre-dexmedetomidine cooling duration, range: 5.5 to 23.8 h). Neonates received dexmedetomidine infusion for an average of 64.8 ± 6.9 h (range: 54.3 to 74 h).

### 3.1. Time Course of Plasma Dexmedetomidine during and after Infusion

**Figure 2:**
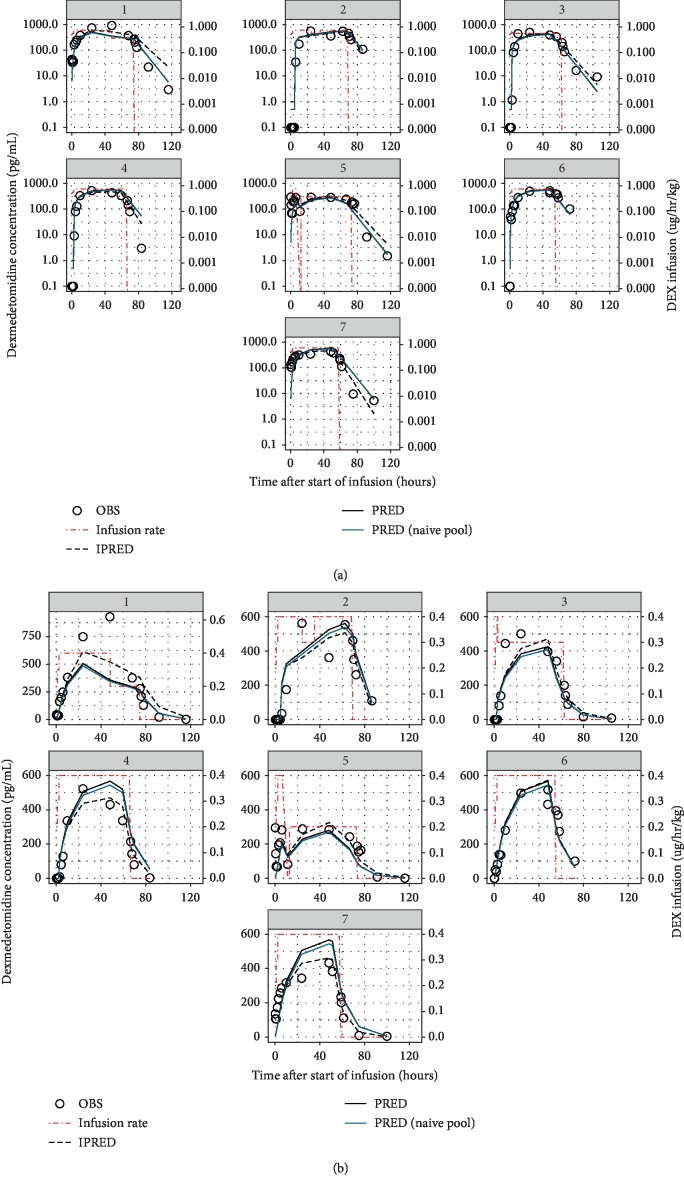
Comparison of the observed plasma dexmedetomidine time course (OBS, open circles) to typical subject prediction (PRED, black solid line) and individual-level prediction (IPRED, black dash line) based on the respective population model. Predictions based on the naïve pooled compartmental model (blue solid line) are overlaid for comparison. Body weight-normalized dexmedetomidine-infused rates, which varied across subjects, are also depicted (red dotted-dash line). Upper and lower panels present the respective semilogarithmic and rectilinear plots; the former is better in showing the model fit to data in the postinfusion phase, whereas the latter shows the fit during infusion more clearly. Variation in the infusion regimen across the subjects is also more evident in the rectilinear plots.

A total of 94 blood samples were collected for dexmedetomidine pharmacokinetic analyses from the 7 neonates with HIE who underwent TH. The time course of observed plasma drug concentration during and after dexmedetomidine infusion for all 7 neonates with HIE is displayed in Figure 2. In 4 of the 7 neonates, dexmedetomidine concentration in plasma remained low or undetectable over the first few hours. In all subjects, plasma concentrations rose gradually; near plateau levels, ranging from 300 to 900 pg/mL, were approached only after 12 to 24 h of infusion (i.e., >10 h after the final step of infusion to 0.4 *μ*g/kg/h at 2.5 h). Upon discontinuation of dexmedetomidine infusion at 6 h after rewarming, plasma dexmedetomidine concentration declined exponentially and remained detectable up to as long as 43 h after infusion ceased. Descriptive pharmacokinetic parameters derived from the noncompartmental analysis are presented in Table 1.

### 3.2. Dexmedetomidine Loss to Infusion Tubing

Due to unexpectedly low plasma concentrations observed during initial hours of dexmedetomidine infusion, mock infusion experiments were conducted to investigate possible drug loss from the intravenous infusion setup. Dexmedetomidine concentrations in samples collected either directly from the syringe or microbore tubing outflow immediately before initiation of infusion or from the syringe at termination of infusion were all close to the nominal concentration of 4 *μ*g/mL; their FNC averaged 0.98 (±0.02). This indicates negligible loss of dexmedetomidine while the infusate was held in the syringe and during quick priming of the microbore tubing.

Loss of dexmedetomidine through sorption to the microbore tubing was observed over the 18 h of infusion (Figure 1). Dexmedetomidine concentration in the outflow infusate declined steadily over the first 6 h; FNC reached a nadir of 0.73 (±0.11). Thereafter, outflow infusate concentration began to recover towards the expected value; by 18 h, FNC had returned to 0.90 (±0.04) when dexmedetomidine sorption apparently became saturated. Linear extrapolation suggests that infusate should attain nominal concentration by 25.1 h. Overall, there was an average 5% cumulative loss of dose delivered over the 54.3 to 74.0 h of dexmedetomidine infusion.

### 3.3. Pharmacokinetic Modeling

A one-compartment model incorporating variable, subject-specific infusion rates adequately described the available pharmacokinetic dataset. The model included adjustments for dexmedetomidine infusion rates for the estimated loss to infusion tubing, censoring due to observations below the lower limit of quantification, and additive residual error. The population model was examined after consideration of a naïve pooled fit; similar pharmacokinetic estimates were obtained under both approaches. The population model included a between-subject variability term on K. Use of the “M3 method” to account for censored PK observations at the start of the infusion (most likely on account of sorptive loss) had a negligible impact; parameter estimates differed by <10% when this approach was not used. For the population fit, the random (variance) components of the model featured an additive residual error parameter and included an exponential between-subject variability in the elimination rate parameter (K). As would be expected for a population model with only seven subjects, the between-subject variability was not estimated with high precision. More complex or alternate variance components (such as an additional, proportional residual error parameter or models with the between-subject variability parameter on CL and/or V) did not appreciably improve goodness of fit; that is, these models resulted in only slightly smaller decreases in the OFV and similar goodness-of-fit diagnostics while exhibiting considerably a higher condition number and large SE of parameter estimates.

As the washout data on a semilogarithmic plot showed convex features in some subjects, we also examined fit of a two-compartment model to the data. The two-compartment model offered only marginal improvements over the one-compartment model counterpart in ΔOFV (−0.8) and goodness-of-fit plots while resulting in a very high condition number (>1000). Under the two-compartment model, dexmedetomidine distribution was predominantly (94%) ascribed to the peripheral compartment (*V*_2_), along with a very fast intercompartmental CL (14 L/h/kg), some 20 times higher than the elimination CL from the central compartment (0.69 L/h/kg). The latter observation indicates near collapse of the two-compartment model to the one-compartment model. Hence, the present set of data does not support the use of a two-compartment model.

We also investigated the possible presence of nonstationarity in dexmedetomidine pharmacokinetics by featuring time-varying model parameters. Per TH protocol body temperature and duration of cooling prior to dexmedetomidine infusion were deemed to represent informative, mechanistic patient-level covariate information, an alternative to the more-commonly employed, empirical, stepwise, linear, or exponential time functions. Inclusion of body temperature as a physiological covariate on CL resulted in a relatively small change in OFV (−2.5) compared to the more empirical stepwise change in CL upon discontinuation of TH (−4.8 ΔOFV) or when CL was assumed to change monoexponentially or linearly after the start of infusion (−13.2 or −14.2 ΔOFV). Incorporation of duration of cooling prior to the start of infusion as a covariate for CL was noted to result in a small OFV decrease (−4.2), and a model exhibiting high SE of the estimate for this parameter. While mechanistically appealing, we cannot conclude that time-varying pharmacokinetics occurred in this small cohort of HIE neonates. Further studies will be required to address this possible complexity of dexmedetomidine pharmacokinetics. At present, the conventional (time-invariant) one-compartment model appears to adequately describe dexmedetomidine pharmacokinetics.

All the demographic or clinical variables: gestational age, postmenstrual age, body weight, serum CrMax, and serum ALT, when incorporated into the final model as covariates of CL resulted in very small decreases in OFV (<3.4). Models with these covariates on V resulted in even smaller ΔOFVs. In view of the limited number of subjects, the small effect size of these covariate estimates, and a generally high condition number of models with two or more covariates, we concluded that none of these patient-specific factors appear to significantly influence dexmedetomidine pharmacokinetics.

Table 2 presents the parameter estimates for both the naïve pooled and population models, along with their absolute and relative standard errors; parameter estimates are similar between the two compartmental modeling approaches. Population estimates of CL and V for the one compartment model, 0.697 L/h/kg and 7.48 L/kg, respectively, are also reasonably consistent with estimates derived from noncompartmental analysis in Table 1. The elimination half-life corresponding to the typical CL and V is 7.3 h.

**Figure 3:**
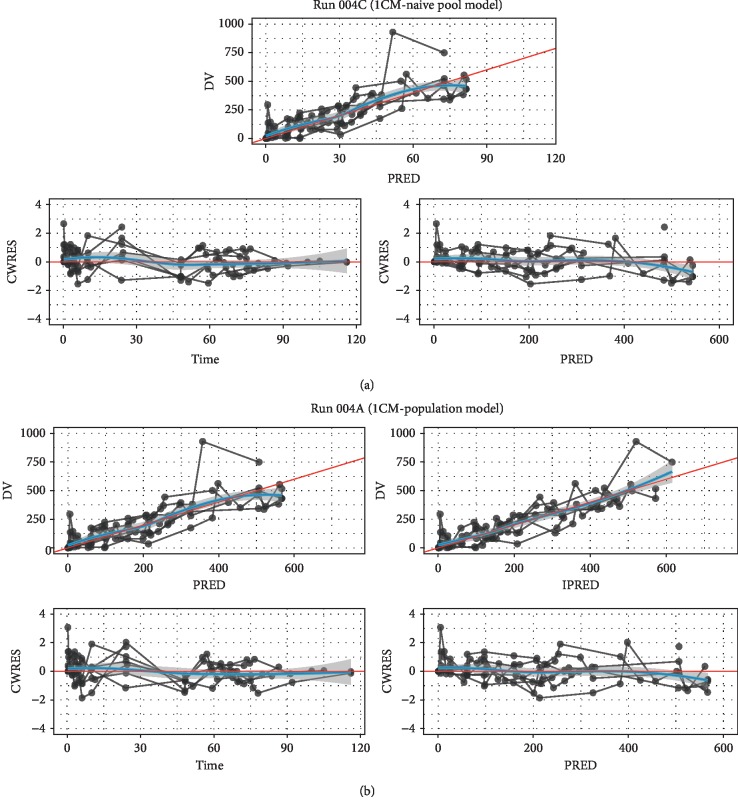
Goodness-of-fit plots for the one-compartment naïve pooled model (a) and population model (b). (a) and (b) depict plots of observed (DV) vs. population prediction (PRED) or individual prediction (IPRED) and plots of conditional weighted residuals (CWRES) vs. time or PRED. PREDs are equal to IPREDs for the case of the naïve pooled model.

**Figure 4:**
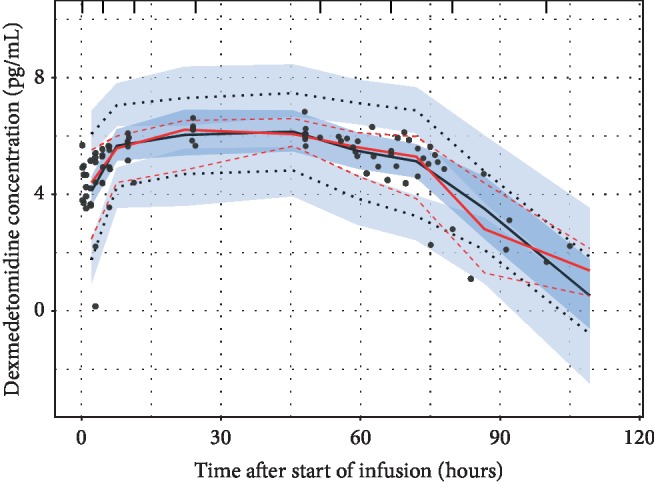
Visual predictive check (VPC) plot of the final dexmedetomidine population model providing a comparison of observed and predicted data. Individual concentrations are depicted using black points, with the median for the observed concentration-time course being depicted as a solid red line and its simulated 5th and 95th percentiles. The median for model predictions is depicted as a black solid line, with corresponding simulated 5th and 95th percentiles being depicted as a dotted black line. The simulated 90% prediction intervals for the 5th, 50th, and 95th levels are depicted using light blue polygons (prediction area overlaps appear as darker polygons).

Figure 2 shows the population (typical subject) and individual-level predictions of the one-compartment model; they describe the observed data well. As expected, the naïve pooled model predictions followed the population predictions quite closely.  Figure 3 compares the standard goodness-of-fit plots between the population and naïve pooled approaches, indicating that only a very modest improvement in goodness of fit was observed for the population model. Observations, typical subject (population-average) predictions and quantiles of the observed and simulated data are presented as a visual predictive check (VPC) in Figure 4, illustrating that the central tendency of the data is well captured with the current population model.

### 3.4. Clinical Characteristics

All enrolled neonates had moderate-to-severe HIE. The mean gestational age was 39 ± 1.3 weeks and mean birth weight was 3501 ± 588 grams. Most of the neonates were of white race (6/7, plus 1 native Hawaiian/Pacific Islander). Over half (4/7; 57%) of neonates were delivered via emergent cesarean section.  Table 3 presents the NICU hospital course characteristics of the study neonates. Five neonates (71%) had a 10-minute APGAR score ≤5. Three (43%) neonates required chest compressions, 2 of whom received epinephrine during newborn resuscitation. Most of the neonates (5/7; 71%) had seizures; 4 had seizures prior to starting dexmedetomidine. One neonate had a single, brief seizure not requiring treatment that occurred 5 h after dexmedetomidine was started; thereafter, no further seizure activity was noted. Four neonates required mechanical ventilation for >12 h, but all the surviving neonates were sent home on room air. Of the 7 neonates in our study, 3 required no inotropic support during dexmedetomidine treatment, and one (patient #2) was weaned off dopamine within 1 h after starting dexmedetomidine. Patient #3, who was titrated off dopamine prior to starting dexmedetomidine, was placed back on dopamine 18 h after starting dexmedetomidine and then was weaned off dopamine 18.5 h later. Patient #5 was placed on dopamine 12 h after starting dexmedetomidine and was weaned off dopamine 27 h later. Patient #1, who had severe HIE with multiorgan failure and was treated with dopamine and epinephrine infusions during dexmedetomidine treatment, continued to require dopamine for 3 additional days after dexmedetomidine was discontinued. This neonate died at 10 days of age after her life support was withdrawn per parental request.

Breakthrough shivering episodes treated with morphine were recorded in 29% (2/6) of neonates during TH. One neonate (patient #4), who had 7 shivering episodes, received 3 total doses of morphine, each for a shivering episode that lasted 5 min. Another neonate (patient #5) had 2 mild shivering episodes (duration not documented) and received a dose of morphine for each episode.

Prior to starting dexmedetomidine, the baseline average N-PASS score ranged between 0 and 2 (63 time points recorded) for patients #2–7 and was −6 (3 time points) for patient #1. During the dexmedetomidine infusion period, 244 N-PASS scores were collected over time. Most neonates were without signs of pain or agitation, with only 3.7% (9/244) of N-PASS scores >3 at any time point. The majority of neonates (85.2%; 208/244 time points) had N-PASS scores between −5 and +3. Of the 11.1% (27/244) of scores between −6 and −10, reflecting deeper sedation levels, most of these (25/27) were accounted for by one neonate with severe global brain injury (patient #1). Patient #7 received 5 morphine doses prior to starting dexmedetomidine and 4 morphine doses while receiving dexmedetomidine during TH for elevated N-PASS scores (≥3). In addition to morphine, lorazepam was used once for placement of a peripherally inserted central catheter (0.05 mg/kg; patient #2) and once for an elevated N-PASS score of 4 (0.1 mg/kg; patient #5). No other benzodiazepines were used while the neonates were receiving dexmedetomidine infusions.

### 3.5. Adverse Events

To assess any study safety concerns, the NICU clinical course of every neonate enrolled in the study was reviewed by the Internal Safety Monitoring Committee during and after study completion. Overall, no adverse events or serious adverse events were associated with dexmedetomidine. No neonates had hypertension or bradycardia episodes associated with dexmedetomidine. Following a dose of lorazepam for placement of a peripherally inserted central catheter, one neonate (patient #2) in our study had a transient bradycardia that prompted a temporary dexmedetomidine rate reduction to 0.3 *μ*g/kg/h; further bradycardic events were not noted after the dexmedetomidine rate was increased back to 0.4 *μ*/kg/h. During dexmedetomidine treatment, three neonates (patients #1, 3, and 5) had hypotension that was responsive to inotropic support. These three neonates did not have hypotension below the cutoff considered to be an adverse event (<32 mm Hg mean arterial pressure) based on the study safety guidelines approved by the Seattle Children's Hospital Institutional Review Board. None of the neonates had atrial fibrillation or cardiorespiratory decompensation. The one neonate who died suffered from severe multiorgan failure and had care withdrawn electively at 10 days of age, per parental request. None of the 7 neonates had positive blood cultures or central line-associated blood stream infections.

## 4. Discussion

This is the first report of a clinical study on the pharmacokinetics of dexmedetomidine in neonates with HIE undergoing TH. The key question raised in this study was whether the clinical complications of HIE and abnormal physiology induced by TH altered dexmedetomidine pharmacokinetics. We identified three reported studies in the literature that allowed for comparison of our data to findings in non-HIE, normothermic newborns.

The most comparable set of dexmedetomidine pharmacokinetic data for normothermic term neonates without HIE was reported by Chrysostomou et al. [15] (see summary in Table 1). This was a phase II/III, open-label, multicenter safety, efficacy, and pharmacokinetic study of dexmedetomidine that included 24 term neonates. Evaluable pharmacokinetic data were available from 13 term neonates; they received 3 escalating dexmedetomidine dose levels, including a loading dose (LD, *μ*g/kg) followed by a maintenance dose (MD, *μ*g/kg/h) for a median of 6 h (range, 6–14.4 h): level 1, 0.05/0.05 LD/MD (*n* = 1); level 2, 0.1/0.1 LD/MD (*n* = 5); and level 3, 0.2/0.2 LD/MD (*n* = 7). Chrysostomou et al. reported their findings based on a noncompartmental pharmacokinetic analysis, which are directly comparable to results of our noncompartmental analysis presented in Table 1. The mean clearance (0.91 ± 0.50 L/h/kg) reported by Chrysostomou et al. [15] for normothermic neonates without HIE of similar gestational and premenstrual age was somewhat higher but not statistically different than the mean CL corrected for the sorptive loss in dose (CL_cor_, 0.76 ± 0.16 L/h/kg) in our cohort of HIE neonates undergoing TH. MRT was shorter (4.26 ± 3.90 vs. 6.84 ± 3.20 h) and *V*_ss_ was smaller (4.57 ± 4.26 vs. 5.22 ± 2.62 L/kg) in the non-HIE, normothermic cohort; again, neither of these differences reached statistical significance, most likely due to the large variability reported in Chrysostomou's study.

Additional dexmedetomidine pharmacokinetic data were extracted from studies by Greenberg et al. [13] and Potts et al. [28] in pediatric populations of broader age range (i.e., including infants and young children). The study by Greenberg et al. was an open-label, single-center pharmacokinetic study of dexmedetomidine involving 20 infants (median gestational age of 39 weeks, range 27–40 weeks; postnatal age of 43 days, range 4–203 days), who received continuous dexmedetomidine intravenous infusions (median 1 *μ*g/kg/h, range 0.1–2.5 *μ*g/kg/h), with additional intravenous bolus doses when needed (median 0.5 *μ*g/kg, range 0.1–3.0 *μ*g/kg) [13]. Potts et al. reported a population pharmacokinetic analysis of pooled plasma concentration-time data derived from four earlier (pre-2009) studies on 95 non-HIE pediatric intensive care patients (mean age of 3.8 years), who received either a dexmedetomidine intravenous bolus (1 *μ*g/kg) or infusion (0.2 *μ*g/kg/h) regimen [28].

**Figure 5:**
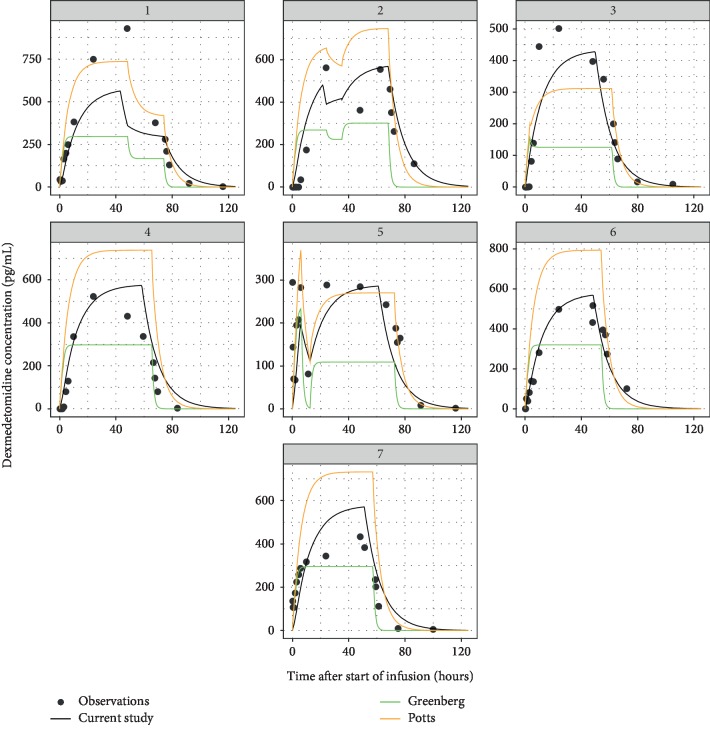
Comparison of individual-level predictions based on the dexmedetomidine population model from the current study (black) and those reported by Greenberg et al. (green) and Potts et al. (orange). Simulations for the Greenberg and Potts models took into account individual-level covariates (postnatal age and body weight) from the present study.

In both studies, the investigators performed population pharmacokinetic modeling using premenstrual age and body weight as covariates to account for the influence of age and development. Hence, their models afforded individual-level predictions for non-HIE, normothermic counterparts (external model “controls”) to our 7 neonatal subjects.  Figure 5 compares predicted plasma concentration-time profiles based on our present population model for the 7 HIE, hypothermic newborns to those based on the population pharmacokinetic models of Greenberg et al. and Potts et al. for the non-HIE, normothermic counterparts. The models of Greenberg et al. and Potts et al. predicted that dexmedetomidine concentration in normothermic neonates without HIE would rise quickly and reasonably in step with the upward titration in dexmedetomidine infusion rate. The plateau plasma concentrations predicted by the Greenberg model were consistently lower than those predicted by our model, whereas predictions by the Potts model were generally higher. Since plateau or steady-state plasma concentration during intravenous infusion is entirely governed by plasma clearance, the above observations are consistent with the comparison of mean corrected CL observed in our cooled neonates (0.761 ± 0.155 L/h/kg) with those predicted for normothermic counterparts based on the population models of Greenberg et al. (vs. 1.23 ± 0.08 L/h/kg, *p* < 0.01) and Potts et al. (vs. 0.548 ± 0.032 L/h/kg, *p*=0.02), as presented in Table 1. The average *C*_max_ predicted by the Greenberg model (304 ± 49 pg/mL) was lower than our observed average by ∼43% (537 ± 180 pg/mL, *p*=0.02), whereas the average *C*_max_ predicted by the Potts model (670 ± 128 pg/mL) was higher but did not reach statistical significance. Both Greenberg et al. and Potts et al. models predicted significantly shorter MRTs (1.24 ± 0.09 and 3.28 ± 0.19 h vs. 6.84 ± 3.20 h, *p* < 0.01 and *p*=0.03, respectively) and smaller *V*_ss_ (1.51 and 1.79 vs. 5.22 ± 2.62, *p* < 0.01 and *p*=0.02, respectively).

Overall, the above analysis suggested some distinction in dexmedetomidine pharmacokinetics between cooled neonates with HIE and normothermic neonates without HIE. Mean dexmedetomidine CL in the current study cohort was comparable to or slightly lower than values for normothermic newborns without HIE reported by Chrysostomou et al. [15] and predicted from the population model of Greenberg et al. [13] (Table 1). In contrast, mean CL for our HIE cohort was higher than that for a corresponding normothermic cohort predicted by the population model of Potts et al. [28]. It should be noted that Potts et al. performed a pooled analysis of earlier literature data collected in mostly older infants (>1 month of age) and young children; there were only 4 neonates out of a study population of 94 subjects. Thus, the Potts population model may be biased towards older infants and children. Therefore, our conclusions should rely largely upon comparisons with the data of Chrysostomou et al. [15] and Greenberg et al. [13]. Our analysis further indicated that HIE newborns undergoing TH have larger distribution volumes and shorter MRTs than anticipated in a comparable cohort of non-HIE, normothermic newborns.

Several caveats are worth noting. First, our analysis is based on a very limited dataset from just 7 subjects; hence, our observations will need to be confirmed by further studies in larger cohorts of HIE newborns. Second, the comparisons with dexmedetomidine pharmacokinetics reported in earlier studies may be biased by study design differences in blood sampling; the present study is exceptional in that washout kinetics of dexmedetomidine after discontinuation of drug infusion was followed for an extended period of 42 h. In all previous studies in normothermic neonates without HIE, blood sampling was limited to a time span of no more than 24 h after either bolus intravenous dose or short-term infusion.

The reason for dexmedetomidine CL tending to be lower in HIE newborns undergoing TH compared to normothermic, non-HIE newborns needs further investigation. Dexmedetomidine is cleared in adult humans almost exclusively by metabolism through direct *N*-glucuronidation and cytochrome P450 metabolism (hydroxylation, mediated by CYP2A6) [30]. Minimal dexmedetomidine dose is recovered in urine or feces as an unchanged drug; hence, renal function is not an important consideration in this context. Depressed hepatic drug metabolism in the HIE newborns undergoing TH could be an explanation for its lower clearance. In this regard, it is interesting to note that Frymoyer et al. reported markedly lower morphine clearance in neonates with HIE receiving hypothermia compared with reports in full-term normothermic neonates <7 days old without HIE [4]. Clearance of morphine, which mainly occurs through UGT2B7-mediated *O*-glucuronidation in the liver, was not correlated with liver injury (based on elevated alanine aminotransferase levels) in their study. We also did not find the liver enzyme marker, serum alanine aminotransferase, to be a significant covariate for dexmedetomidine clearance in our population modeling.

In practical terms, our current study suggests that the standard dexmedetomidine maintenance infusion rates recommended for typical newborns in an intensive care setting may be reasonably appropriate for neonates with HIE undergoing TH, at least with respect to achieving similar or slightly higher steady-state plasma drug concentrations. Our study does suggest that a loading dose may be needed to overcome the initial lag in achieving effective plasma levels of dexmedetomidine due to a longer elimination half-life in HIE newborns; the elimination half-life predicted by the compartmental model is about 7.3 hours (i.e., estimated from CL and V reported in Table 2), which is longer than the generally accepted value of 3 hours for normothermic, non-HIE neonates. This means dexmedetomidine steady state would not be achieved until about 28 hours (i.e., 4 half-lives) after initiation of or change in infusion rate in HIE neonates undergoing TH compared to only 13 hours in normothermic, non-HIE neonates. The delay in achieving an infusion steady state may be further complicated by drug loss through sorption to intravenous microbore tubing; the latter could result in as much as 30% lower actual infusion rate than intended during the first 6 hours of infusion (Figure 3). Sorptive loss of dexmedetomidine to plastic intravenous tubing, which effectively lowers the dose delivered, is probably dependent on the particular infusion set; this potential issue should always be considered when patients treated with standard doses of dexmedetomidine do not respond with the anticipated sedative, analgesic, anxiolytic, and antishivering effects in a timely fashion. While a loading dose may lead to a faster attainment of steady-state level, the current dosing regimen appeared to provide effective sedation and appeared safe, so a bolus dose should probably be reserved for cases where sedation or preventing shivering is ineffective.

The neonates in the current study were critically ill, evidenced by their severe metabolic acidosis with a mean pH of 6.90 and base deficit of 22 after birth. In addition to neonatal encephalopathy related to moderate-to-severe HIE, the majority of neonates had seizures and elevated alanine aminotransferase levels, required mechanical ventilation, and needed inotropic support for hypotension. Although it is unclear if dexmedetomidine treatment resulted in the need for inotropic support, clinicians considering dexmedetomidine treatment for neonates with HIE during TH should closely monitor hypotension as a potential side effect. Despite their clinical lability, titration of intravenous dexmedetomidine infusion up to 0.4 *μ*g/kg/h was well tolerated, without apparent adverse events. Based on N-PASS scores, the neonates had adequate sedation with dexmedetomidine for the majority of the study, with only one neonate (patient #1, who had severe global brain injury) being potentially oversedated, but that neonate also had an N-PASS score of −6 prior to starting dexmedetomidine. The predetermined maximum study dosage was much lower than dexmedetomidine doses commonly used in critically ill neonates in the Seattle Children's NICU (e.g., doses up to 1.2 *μ*g/kg/h; unpublished data); this maximum dosage of 0.4 *μ*g/kg/h was chosen to avoid potential adverse side effects since neonates with HIE often have multiorgan failure.

Small sample size is a limitation of our present study. The current study was designed to assess the pharmacokinetics and safety of dexmedetomidine in neonates with HIE treated with TH and was not powered to evaluate dexmedetomidine efficacy, and therefore, any safety-related results should be carefully considered. Only one neonate had multiorgan dysfunction, so our ability to describe the pharmacokinetic profile and clinical response to dexmedetomidine in the setting of multiorgan dysfunction is limited. Despite a limited number of subjects, a total of 94 pharmacokinetic samples were collected from the 7 neonates with HIE who underwent TH, which are comparable to the total samples collected from 20 infants in the study by Greenberg et al. (*n* = 89) [13]. We believe that rich sampling in select subjects is preferable to sparse sampling in a larger number of subjects, given the unknown effects of TH on dexmedetomidine pharmacokinetics prior to this study. We could not have discerned the time-dependent kinetics of dexmedetomidine in our HIE cohort undergoing TH if our study had followed a sparse blood sampling design.

## 5. Conclusions

Differences in dexmedetomidine pharmacokinetics were observed in the current small cohort of newborns with HIE undergoing TH compared to reported literature data for normothermic neonates without HIE. Dexmedetomidine clearance in our 7 HIE neonates is comparable to or lower than reported clearance in normothermic, non-HIE neonates. Our HIE newborns also exhibited a larger volume distribution and a longer mean residence time or elimination half-lives compared to their non-HIE counterparts. There was a notable delay or slow initial rise in plasma drug concentration upon dexmedetomidine infusion due to a combination of longer elimination half-life and sorptive loss during delivery through the microbore infusion tubing. These findings suggest that, in cooled neonates with HIE, a loading dose or more rapid escalation in initial titration of dexmedetomidine infusion may be needed in order to achieve effective levels in a timely manner. Safety is suggested by our data; however, considering the limitations of our small sample size and lack of a control group, further evaluation of dexmedetomidine in neonates with HIE treated with TH is warranted. A larger, well-powered trial is needed to determine the effectiveness of dexmedetomidine vs. morphine at providing sedation and preventing shivering and to elucidate any long-term neurodevelopmental impact in neonates with HIE treated with TH.

## Figures and Tables

**Table 1:** Comparison of dexmedetomidine pharmacokinetics in 7 neonates with HIE undergoing TH to literature data in normothermic, non-HIE neonates.

	Subject #							Present	Chrysostomou [15]	Greenberg [13]	Potts [28]
	1	2	3	4	5	6	7	Mean ± SD or median (range)			
Subject characteristics											
Gestational age (wks; days)	39 5 d	41 5 d	37 3 d	40 4 d	40 6 d	38 0 d	39 0 d	39.6 ± 1.4	39.1 ± 1.6	39 (27–40)	NR
Postnatal age (d, wks, or yrs)								1.7 ± 0.5 d	2.23 ± 1.60 wks	6.14 (0.57–29) wks	3.8 (0.02–14) yrs
Body weight (kg)	3.83	3.47	2.41	3.84	3.08	4.13	3.81	3.51 ± 0.54	3.40 ± 0.60	4.02 (2.00–6.00)	16.1 (3.1–58.9)
Time from birth to TH (h)	4.55	4.78	4.60	8.50	3.65	5.78	4.7	5.22 ± 1.57	NA	NA	NA

Dexmedetomidine dosing											
Time from TH to infusion start (h)	4	9.7	16.2	12.5	5.5	23.7	20.7	13.19 ± 7.44	NA	NA	NA
Duration of infusion (h)	74.0	68.3	61.8	65.5	72.5	54.3	57.3	64.8 ± 6.9	6–24	Variable	Bolus or 8 h inf.
Maintenance infusion rate (*μ*g/kg/h)	0.4	0.4	0.4	0.4	0.4	0.4	0.4	0.4	0.2	0.5–2.5 (max)	0.4 (under age 1 yr)
Nominal infused dose (*μ*g/kg)	24.1	25.9	18.5	25.9	14.2	21.4	22.6	21.8 ± 3.9	NR	NR	NR
Corrected infused dose (*μ*g/kg)	22.8	25.0	17.8	24.9	13.5	19.8	21.1	20.7 ± 3.8	NA	NA	NA

Descriptive PK parameters											
*C*_max_ (pg/mL)	929	562	501	523	295	517	433	537 ± 180	968 ± 1011^a^	304 ± 49^b,^^*∗*^	670 ± 128^b^
*T*_max_ (h)	48.0	24.0	24.0	24.0	0.2	48.3	48.3	31.0 ± 16.8	NR	End of infusion	End of infusion
AUC_0–∞_ (ng/mL·h)	47.4	32.3	25.5	26.0	19.5	26.4	21.8	28.4 ± 8.6	NR	NR	NR
CL (L/h/kg)	0.480	0.775	0.699	0.958	0.694	0.752	0.968	0.761 ± 0.155	0.907 ± 0.502	1.23 ± 0.08^*∗∗*^	0.548 ± 0.032^*∗*^
MRT (h)	4.05	11.4	4.73	2.64	6.85	11.3	6.79	6.84 ± 3.20	4.26 ± 3.90	1.24 ± 0.09^*∗∗*^	3.28 ± 0.19^*∗*^
*V*_ss_ (L/kg)	1.95	8.88	3.31	2.53	4.75	8.54	6.57	5.22 ± 2.62	4.57 ± 4.26	1.51^c,†^	1.79^d,†^

Abbreviations: NR, not reported; NA, not applicable or available; TH, therapeutic hypothermia (33.5°C); PK, pharmacokinetics; *C*_max_, maximum observed plasma concentration; *T*_max_, time of *C*_max_; AUC_0–∞_, area under the plasma concentration-time curve from time 0 to infinity; CL, clearance based upon the infused dose corrected for sorptive loss; MRT, mean residence time; *V*_ss_, steady-state distribution volume. Note that, for all the pharmacokinetic parameters, estimates presented in the Potts et al. and Greenberg et al. columns are mean ± SD for the 7 normothermic counterparts predicted based upon the reported population pharmacokinetic models. ^a^Extrapolated from an infusion rate of 0.2 *μ*g/kg/h, i.e., multiplying reported mean ± SD by 2. ^b^Average of simulations for normothermic counterparts to each of our 7 cooled newborns without HIE at the maximum infusion rate of 0.4 *μ*g/kg/h. ^c^Greenberg et al. were only able to provide the population mean estimate of distribution volume because of insufficient data in the initial accumulation or washout phases of DEX infusion, i.e., no modeling of interindividual variation. ^d^Distribution volume was scaled to body weight; hence, volume per kg did not differ between the normothermic counterparts. ^*∗*^*p* < 0.05 for a 2-tailed, 2-sample *t*-test between reported values for normothermic, non-HIE newborns and presently observed values for cooled newborns with HIE. ^*∗∗*^*p* < 0.01 for a 2-tailed, 2-sample *t*-test between reported values for normothermic, non-HIE newborns and presently observed values for cooled newborns with HIE. ^†^*p* < 0.02 for a 2-tailed, one-sample *t*-test between the reported fixed value for normothermic, non-HIE newborns and presently observed values for cooled newborns with HIE.

**Table 2:** Parameter estimates, along with their standard error and relative standard error, for the naïve pooled model and the population one-compartment model.

Parameter	Estimate	Standard error	Relative standard error
Naïve pooled model			
Clearance (CL) (L/h/kg)	0.726	0.0253	0.0349
Volume of distribution (V) (L/kg)	7.90	1.05	0.133
Additive residual error (pg/mL)	109	6.86	0.0630

Population model			
Clearance (CL) (L/h/kg)	0.697	0.0869	0.125
Volume of distribution (V) (L/kg)	7.48	1.17	0.157
Additive residual error (pg/mL)	94.3	8.83	0.0936
Between-subject variance on K	0.0394	0.116	2.95

**Table 3:** Hospital course characteristics of neonates with hypoxic-ischemic encephalopathy treated with dexmedetomidine during hypothermia.

Characteristics	1	2	3	4	5	6	7	Mean or no.	SD (±)
Female, *n* (%)	F	M	M	F	M	M	M		
APGAR									
5 min	4	6	0	2	4	0	4	3	2
10 min	4	7	0	3	4	5	8	4	3
Resuscitation									
Chest compressions	Yes	No	Yes	No	No	Yes	No		
Intubation after birth	Yes	Yes	Yes	Yes	Yes	Yes	Yes		
Epinephrine (bolus number)	No	No	Yes (7)	No	No	Yes (1)	No		
Normal saline (bolus number)	No	No	Yes (2)	No	No	Yes (1)	No		
First arterial or capillary pH	6.65	6.98	7.04	6.98	7.06	6.7	6.9	6.90	0.16
Base deficit, mmol/L	34	18	23	17	17	27	19	22	6
Admission rectal temp. (Celsius)	30.9	35	33.6	34.7	34.5	34.5	34.7	34.0	1.4
CrMax, mg/dL	3.7	1.1	0.5	0.8	1.4	1.9	0.8	1.5	1.1
Lowest platelet level	95	201	106	234	75	62	97	124	66
ALTmax, U/L	482	439	127	160	583	129	28	278	217
Highest INR	3	2.1	2.6	1.3	1.8	1.8	1.1	2.0	0.7
Lowest fibrinogen	110	273	62	185	169	118	397	188	114
Intubation > 12 h, *n* (%)	Yes	No	Yes	No	Yes	Yes	No	4/7 (57%)	
Clinical seizure, *n* (%)	Yes	No	Yes	Yes	Yes	Yes	No	5/7 (71%)	
Anticonvulsants	Yes (P, F, Lev)	No	Yes (P, Lev)	Yes (P)	No	No	No	3/7 (43%)	
Inotropic support, *n* (%)	Yes (Dop, Epi, HC)	Yes (Dop)	Yes (Dop, HC)	No	Yes (Dop)	Yes (Dop)	No	5/7 (71%)	
Breakthrough shivering	No	No	No	Yes, X7 (2 sev, 2 mod, 2 mild)	Yes, X2 (mild)	No	NR	2/6 (33%)	
Duration				1–5 min	NR				
Morphine doses given	0	0	0	3	2	0	0		
Bradycardia (<70 bpm)	No	1 episode	No	No	No	No	No	1/7 (14%)	
Death during hospitalization, *n* (%)	Yes	No	No	No	No	No	No	1/7 (14%)	

SD, standard deviation; CrMax, maximum creatinine; ALTmax, maximum alanine aminotransferase; INR, international normalized ratio; P, phenobarbital; F, fosphenytoin; Lev, levetiracetam; Dop, dopamine; Epi, epinephrine; HC, hydrocortisone; NR, not reported.

## Data Availability

The data used to support the findings of this study are available from the corresponding author upon request.

## Supplementary Materials

Supplementary Materials Supplementary Table S1: optimized mass spectrometry parameters for analytes and dexmedetomidine.
