# Supplementary material for: Dexmedetomidine Pharmacokinetics in Neonates with Hypoxic-Ischemic Encephalopathy Receiving Hypothermia
Source: Anesthesiol Res Pract. 2020 Feb 25;2020:2582965. doi: 10.1155/2020/2582965 (PMC7060842; doi:10.1155/2020/2582965)
Supplement: Supplementary Materials — Supplementary Table S1: optimized mass spectrometry parameters for analytes and dexmedetomidine. [file 2582965.f1.pdf]

Supplementary Table S1. Optimized mass spectrometer parameters for analytes

Analyte	Precursor Ion	Secondary Ion	Declustering Potential (volts)	Dwell (ms)	Collision Energy (volts)	Cell Exit (volts)
Dexmedetomidine	201.0	95.0	61	75	23	12
Medetomidine-d_3	204.0	98.0	61	75	23	12
